# Making and Maintaining Lifestyle Changes with the Support of a Lay Health Advisor: Longitudinal Qualitative Study of Health Trainer Services in Northern England

**DOI:** 10.1371/journal.pone.0094749

**Published:** 2014-05-06

**Authors:** Shelina Visram, Charlotte Clarke, Martin White

**Affiliations:** 1 Centre for Public Policy and Health, Durham University, Stockton-on-Tees, United Kingdom; 2 Fuse (UKCRC Centre for Translational Research in Public Health), Newcastle University, Newcastle-upon-Tyne, United Kingdom; 3 School of Health in Social Science, University of Edinburgh, Edinburgh, United Kingdom; 4 Institute of Health & Society, Newcastle University, Newcastle-upon-Tyne, United Kingdom; University of St Andrews, United Kingdom

## Abstract

**Objective:**

To explore and document the experiences of those receiving support from a lay health trainer, in order to inform the optimisation and evaluation of such interventions.

**Design:**

Longitudinal qualitative study with up to four serial interviews conducted over 12 months. Interviews were transcribed and analysed using the constant comparative approach associated with grounded theory.

**Participants:**

13 health trainers, 5 managers and 26 clients.

**Setting:**

Three health trainer services targeting disadvantaged communities in northern England.

**Results:**

The final dataset comprised 116 interviews (88 with clients and 28 with staff). Discussions with health trainers and managers revealed a high degree of heterogeneity between the local services in terms of their primary aims and activities. However, these were found to converge over time. There was agreement that health trainer interventions are generally ‘person-centred’ in terms of being tailored to the needs of individual clients. This led to a range of self-reported outcomes, including behaviour changes, physical health improvements and increased social activity. Factors impacting on the maintenance of lifestyle changes included the cost and timing of health-promoting activities, ill-health or low mood. Participants perceived a need for ongoing access to low cost facilities to ensure that any lifestyle changes can be maintained in the longer term.

**Conclusions:**

Health trainers may be successful in terms of supporting people from socio-economically disadvantaged communities to make positive lifestyle changes, as well as achieving other health-related outcomes. This is not a ‘one-size-fits-all’ approach; commissioners and providers should select the intervention models that best meet the needs of their local populations. By delivering holistic interventions that address multiple lifestyle risks and incorporate relapse prevention strategies, health trainers could potentially have a significant impact on health inequalities. However, rigorous, formal outcome and economic evaluation of the range of health trainer delivery models is needed.

## Introduction

Health trainers were introduced by the UK Department of Health in 2004 and implemented gradually across England from 2005, with the goal of reducing social inequalities in health [1], [2]. The role involves targeting people from marginalised or socio-economically disadvantaged communities and supporting them to increase healthier behaviours, using a ‘support from next door, rather than advice from on high’ approach, based on the principles of peer support [1]. An additional aim is to combat worklessness and social exclusion by providing opportunities for community members to obtain skills and employment in the health sector. By seeking to recruit health trainers from their target communities, the initiative builds on Wanless's vision of a ‘fully engaged scenario’ and also forms part of the Big Society policy idea to encourage local people to take control over their lives [3], [4]. Furthermore, by recruiting a low paid workforce (NHS Agenda for Change band 2 or 3), health trainers represent a potentially cost-effective solution to contemporary public health problems. Peer or lay health advisors have the potential to address key issues such as the need to enhance the equity of service provision, provide culturally appropriate health advice, and care for growing populations with chronic illness [5], [6]. However, concerns have been raised about the hidden costs associated with coordination, training and supervision for these roles, the potential for higher absence and turnover rates, and whether or not they provide added value above expanding existing workforces [7]–[9].

The largely grass-roots development of the health trainer role has resulted in a diversity of local delivery models, each of which has some grounding in the theoretical and empirical literature. In many areas, health trainers are recruited principally for their interpersonal skills and local knowledge, rather than previous work experience or qualifications. The national training programme (a level 2 RSPH award in Understanding Health Improvement and level 3 City & Guilds Certificate for Health Trainers) draws on the extensive literature from health psychology, as well as community development, social support and natural helping [10], [11]. Health trainers tend to work with clients on a one-to-one basis for 6–12 weeks and use theory-based strategies to help people set achievable goals, make concrete plans to attain those goals, and use self-monitoring techniques to improve confidence [12]–[14]. The latter is particularly important in interventions targeting low income groups, who often have a lower confidence and skill base [15]. Demographic and behavioural similarity between intervention providers and recipients has been shown to help in facilitating behaviour change, and this is part of the rationale for recruiting community members as health trainers [16]. There are also clear policy imperatives for providing capacity-building opportunities in areas that tend to be characterised by high levels of unemployment and reliance on state benefits. These opportunities can impact directly on income, skills and personal satisfaction levels, as well as having indirect impacts on health and wellbeing [17]. Health trainers, service users, their families and communities are therefore all potential beneficiaries of the initiative.

Empirical evidence relating specifically to health trainers is limited. Systematic reviews have found promising benefits in the use of similar lay health advisors (LHAs) to improve outcomes for selected conditions, including diagnosis and treatment of acute respiratory infection in children, improving back pain and type 2 diabetes management, and uptake of certain health-promoting behaviours, including smoking cessation, childhood and adult immunisation, breast cancer screening and HIV prevention [18]–[20]. However, there remains insufficient evidence to determine which LHA strategies are likely to be most effective or cost-effective. There is also a dearth of research exploring service user views and experiences, particularly in a UK context. Such evidence will provide valuable insights into the needs of disadvantaged communities and inform the development of tailored interventions, which can then be formally evaluated. We aimed to conduct an in-depth exploration of the health trainer role, including the strategies used to engage with local people and support them to make and maintain lifestyle changes, in order to provide evidence to inform the optimisation and evaluation of health trainer interventions [21]. This aim was met by undertaking a longitudinal qualitative study of three contrasting health trainer services in northern England, involving serial interviews with clients and staff conducted between June 2008 and January 2011.

## Methods

### Ethics statement

Ethical approval was obtained from Newcastle and North Tyneside-1 NHS research ethics committee (09/H0906/17) and governance approval was obtained from the relevant NHS trusts (not named here to preserve the anonymity of the participating organisations and individuals).

All participants were asked for their written, informed consent to take part in the study, have the discussions audio-recorded and for the (anonymised) information to be used in publications. The study information sheet and consent form were read aloud to two visually impaired clients, who were asked to give audio-recorded verbal consent. Due to the longitudinal study design, consent was seen as an ongoing process rather than a one-off event and all participants were asked to provide verbal consent at each follow-up interview.

### Sampling and recruitment

**Figure 1:**
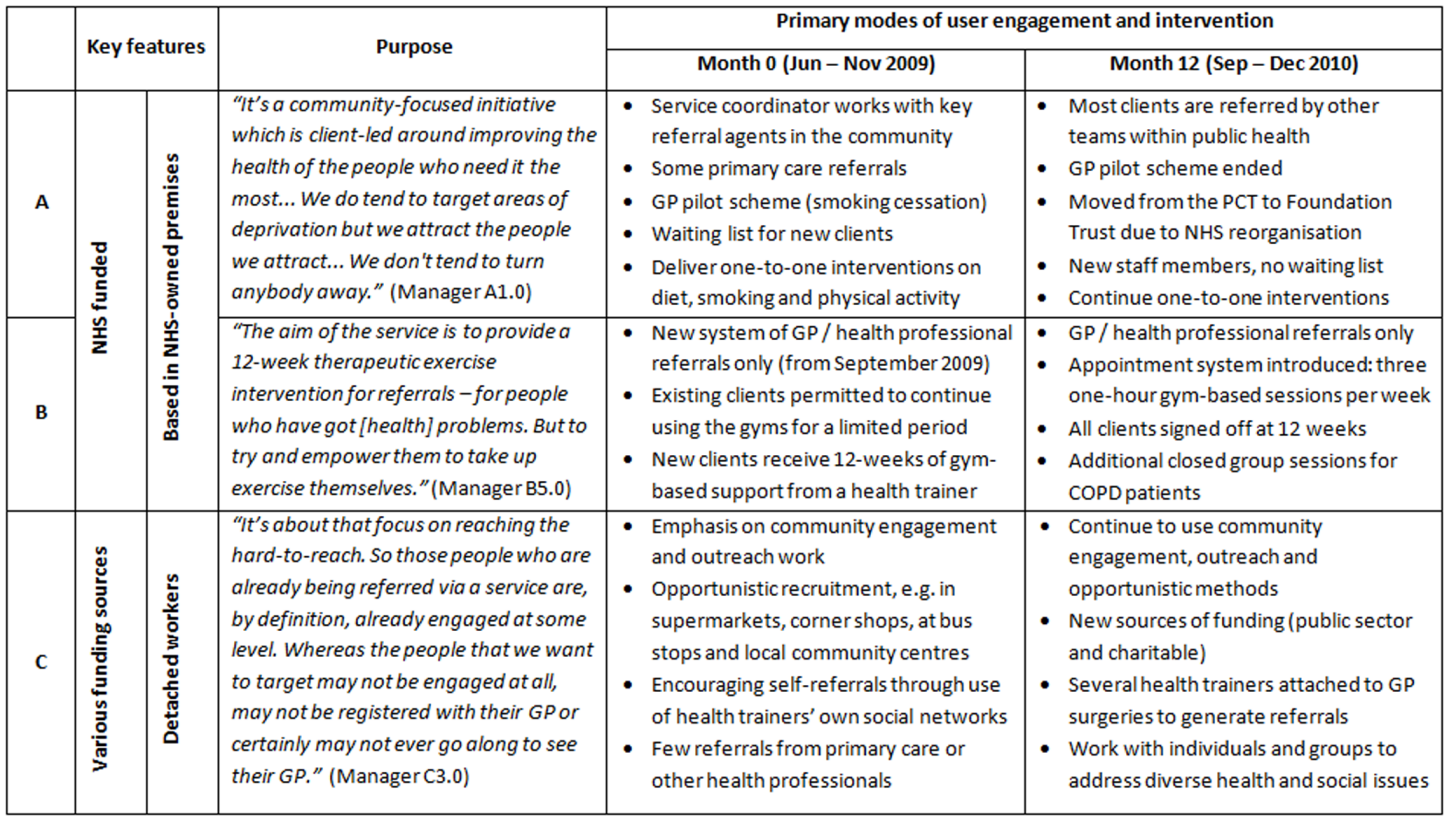
Overview of local health trainer models. Research sites (A, B and C) were chosen to reflect the heterogeneity in local delivery models. The sites are not named to preserve the participants' anonymity.

Three health trainer services were selected to reflect the heterogeneity in local delivery models, based on a previous mapping exercise of the nine health trainer services in north east England (figure 1) [22]. This offered an opportunity to enhance understanding of the underlying processes by which different models influence outcomes. Service A involved NHS health trainers located in various community settings to deliver one-to-one support in relation to diet, smoking and physical activity; service B involved NHS health trainers based primarily in community gyms to deliver an exercise-on-referral intervention; service C involved community health trainers employed within the third sector to deliver a range of one-to-one and group activities.

We recruited health trainers, their managers and clients to the study, in order to obtain an understanding of the various domains of change – individual, organisational, temporal and spatial – from both the service user and provider perspective [23]. After an email introduction by the regional programme lead, the managers of the three services (n = 5) consented to take part in the study (*convenience sampling*). Health trainers (n = 13) were notified about the study during team meetings and then selected using key criteria, such as host organisation, length of time in post, and gender (*purposive sampling*). Recruitment of clients (n = 26) took place via the health trainers and the inclusion criteria were adapted over time to develop emerging theory (*theoretical sampling*). Exclusion criteria included being under 18 years of age, inability to give informed consent, and being employed as a health trainer but not operational at the time of the study. It was made clear that there would be no penalty for those who decided not to take part or withdraw from the study at any time, and this was reiterated at each interview to reduce the possibility of coercion.

### Data generation

**Figure 2:**
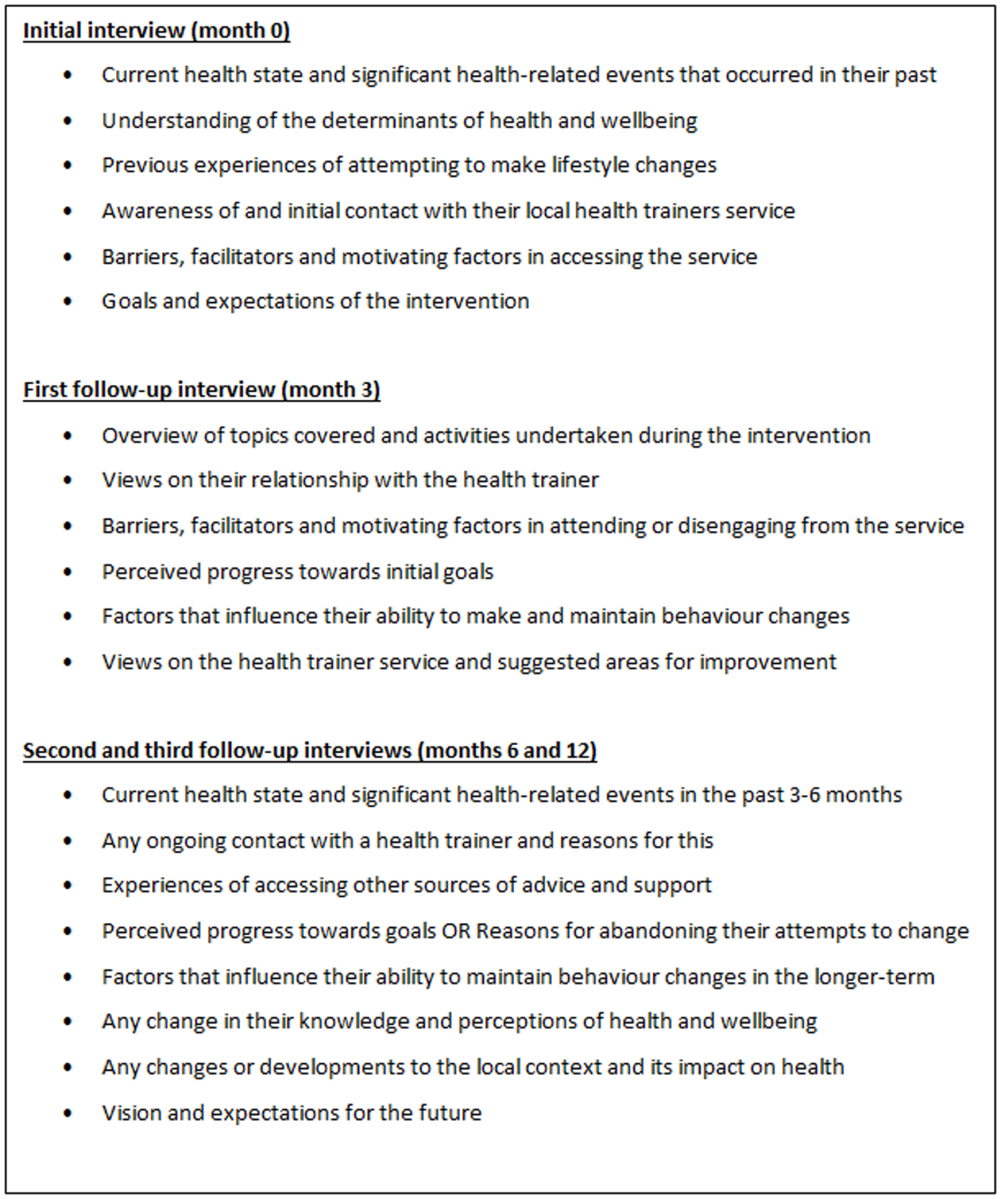
Interview topic guides – clients.

**Figure 3:**
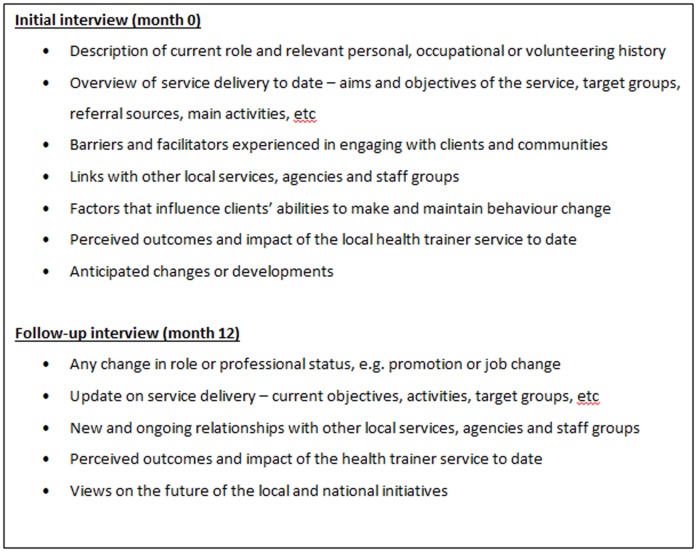
Interview topic guides – staff.

Serial interviews were chosen to explore each client's journey from engaging with a health trainer to maintaining any lifestyle changes following completion of the intervention. The client interviews were undertaken as soon as possible after their first session with a health trainer and approximately three, six and 12 months later, to capture the barriers and facilitators to maintaining lifestyle changes in the short- and medium-term. Interviews with health trainers and managers took place at the beginning of the study and 12 months later, to explore their changing experiences of working with local communities and organisations. Interview topic guides were developed, based on findings from the empirical literature on similar LHA roles and modified over time to focus attention on areas of particular importance (figures 2 and 3). Clients were made aware that their health trainers were being interviewed, that the purpose of these interviews was not to discuss individual clients and that all responses would remain confidential. All staff chose to be interviewed within their workplaces, whereas most clients (n = 20) chose to be interviewed at home. Other mutually convenient venues included a community centre, public library and university building. Detailed field notes were written after each interview, incorporating observations on the environment, actions of the participants, their appearance, non-verbal cues, and any emotions expressed. The notes were used to supplement the interview transcripts (total n = 116) during the data analytical process.

### Data analysis

The interviews were transcribed verbatim, with all identifying information removed. Transcripts and field notes were then analysed using the constant comparative approach associated with grounded theory [24], [25]. This involved conducting the processes of sampling, data generation and analysis iteratively, in order to make the emerging theoretical categories more precise, explanatory and predictive [26]. Data were analysed cross-sectionally to build a picture of each health trainer service at different points in time, and also longitudinally to examine the narratives of each client. We sought specific evidence of behaviour change, but remained open to new and disconfirming information. Line-by-line coding was used to ensure that we stayed close to the data, rather than basing codes on preconceived assumptions [24], [26]. All transcripts and notes were coded manually (by SV) to create a sensitive and nuanced analysis, with NVivo qualitative analysis software (Version 8, Qualitative Solutions and Research) used to store and refine the codes. In the creation of these codes, we were able to move beyond description to micro-analysis of the data. We then began to identify common properties and make comparisons between the concepts. This process was complete once data saturation had been reached, i.e. the point where the initial ‘working’ theory was consistent with all additional data [25].

Regular meetings between the authors led to agreement and confirmation of the emerging themes. Trustworthiness of data interpretation was addressed by CC and MW independently analysing a selection of transcripts. A sub-set of analyses was also shared with a colleague who had no connection to the study, in order to ensure neutrality and openness to unexpected information. The longitudinal design made it possible to re-visit and verify emerging concepts, and the constant comparative approach meant that participants' stories largely guided the inquiry process, thereby enhancing the credibility of the study [27].

## Results

### Participants

**Figure 4:**
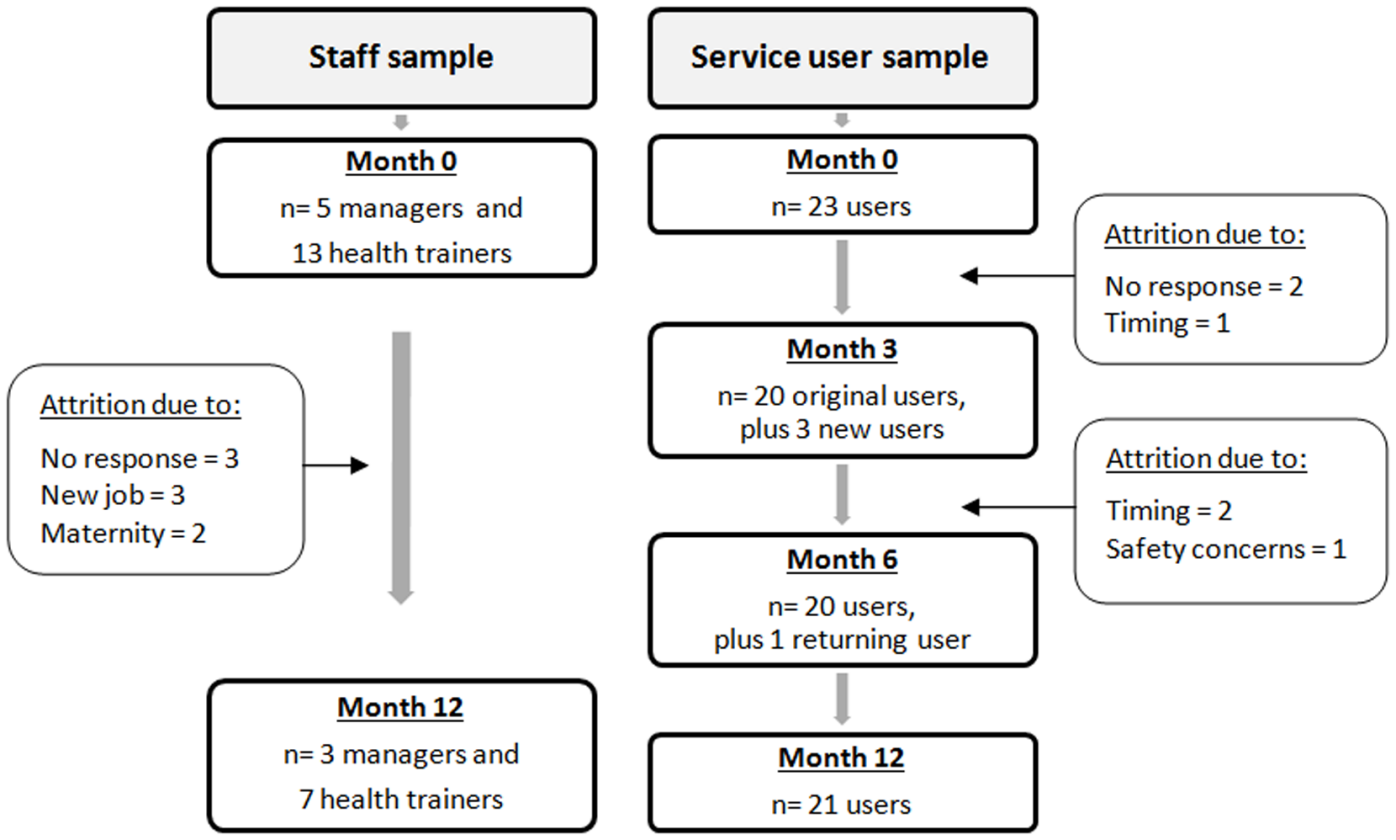
Study flowchart. Sample sizes during each data collection period and reasons for any loss-to-follow-up. ‘Timing’ refers to participants being ill or recently having given birth, while ‘safety concerns’ refers to a participant being excluded from the study after repeatedly asking the researcher instrusive personal questions.

The characteristics of the 44 individuals who took part in the study are given in tables 1 and 2. The majority of clients were female, white European and over the age of 50 years. Figure 4 gives details of the service user and staff sample sizes at each wave of data collection, along with factors contributing to sample attrition.

**Table 1:** Features of the client sample.

Characteristics	Service A (n = 6)	Service B (n = 10)	Service C (n = 10)	Totals (n = 26)
**Age group:**				
Under 30	0	0	1	**1**
30–39	0	0	2	**2**
40–49	1	1	4	**6**
50–59	1	1	1	**3**
60–69	2	6	1	**9**
70–79	2	2	0	**4**
80+	0	0	1	**1**
**Sex:**				
Female	5	5	8	**18**
Male	1	5	2	**8**
**Ethnicity:**				
White European	6	9	9	**24**
British Asian	0	0	1	**1**
Mixed (White & Asian)	0	1	0	**1**
**Employment status:**				
Retired	3	7	2	**12**
Employed	1	1	5	**7**
Long-term unemployed	2	2	2	**1**
Homemaker	0	0	1	**1**

**Table 2:** Features of the staff sample.

Characteristics	Service A (n = 4)	Service B (n = 5)	Service C (n = 9)	Totals (n = 18)
**Role:**				
Health trainer	2	4	7	**13**
Manager	2	1	2	**5**
**Sex:**				
Female	3	3	7	**13**
Male	1	2	2	**5**
**Time in post:**				
Less than 1 year	1	0	3	**4**
1–3 years	3	3	2	**8**
More than 3 years	0	2	4	**6**

### Overview of findings

The use of grounded theory gave participants the freedom to discuss their own priorities, which, for clients, initially centred on historical and contextual factors they felt had contributed to their health concerns. There exists a large body of literature on the social determinants of health and health inequalities (see Bambra et al. [28], McIntyre et al. [29] and Popay et al. [30] for examples) and therefore these factors will not be reiterated here. Furthermore, there is a growing body of literature on the experiences of health trainers and similar LHAs [31]–[33]. Here, we focus on comparing and contrasting the local health trainer services, highlighting the outcomes reported by service users, and identifying factors perceived to influence the longer-term maintenance of these changes. Direct quotations from the participants illustrate these findings, with identifiers used to preserve anonymity (table 3).

**Table 3:** Participant Identifiers.

Sample	Criteria	Examples
Clients	Role: client	Client A1.3 = client no. 1 (of 26),
	Service: A, B, C	service A, month 3
	Participant ID: 1–26	Client B2.6 = client no. 2 (of 26),
	Month of interview: 0, 3, 6, 12	service B, month 6
Staff	Role: health trainer or manager	Health trainer C3.0 = health trainer no.
	Service: A, B, C	3 (of 13), service C, month 0
	Participant ID: 1–13	Manager A1.12 = manager no. 1 (of 5),
	Month of interview: 0 or 12	service A, month 12

### Defining features of health trainer services

Participants across the three research sites outlined features that were perceived to characterise health trainer services. A term used frequently was ‘person-centred’, referring to interventions that were felt to be tailored to suit the needs and circumstances of individual clients. The first meeting between a health trainer and client was seen as crucial in making an initial judgement about the type and intensity of support required. The content of this meeting varied between the three services. Users who had entered via formal referral routes (e.g. via a GP or dietician) were reported to be comfortable with completing paperwork at the first meeting, whereas clients entering via community outreach activities often required time to build trust and develop rapport. Health trainers then worked with clients to determine their priorities, which may or may not have been directly health-related. Diet, physical activity and smoking were identified as primary concerns, but a range of underlying issues often needed to be addressed with those living in disadvantaged areas:

*You have to start from [their] base, don't you? So you're not going to look at your nutrition if you're living in a house that's falling to bits.* (Health trainer C2.0)

There was heterogeneity between the services in the extent to which they were prepared to address these broader issues internally. This appeared to be linked to the host organisation; the NHS-employed health trainers in services A and B were encouraged to signpost ‘complex’ clients to appropriate professional support, whereas service C (in the third sector) dealt with these issues, as far as possible, using holistic, community-based approaches:

*I'm working with a young man at the moment who is struggling to find work… Part of my health trainer job is to get out there in the field of looking for employment for him. […] So everything starts to fit into place. Like, he's on his fitness programme, he's losing his weight, his confidence is building up for him to go out and look for work.* (Health trainer C1.12)

Over time there had been some convergence between the delivery models employed by the three health trainer services (figure 1). For example, there was a general shift towards encouraging GP referrals, as opposed to more opportunistic engagement methods. This was partly motivated by changes in funding, but also by growing awareness and acceptance of the health trainer role among health professionals. All three services were involved in delivering one-to-one interventions based on theory-linked behaviour change techniques set out in the Health Trainer Handbook [10], such as goal setting, barrier identification, behavioural contracting and positive reinforcement:

*[Clients] will come in with these wild and wonderful ideas and I'll go, “Right, now let's get real, you know. Sorry to break your bubble, but it has to start off this way. This is what I can do for you. And I promise you I will, if you promise for me.” And that's the way it works. There's no rocket science over it. It's just down-to-earth, simple action plans. Life action plans and, you know, how these changes can fit into their lifestyle.* (Health trainer C1.0)

Health trainers were able to tailor these interventions and techniques to specific client needs and organisational requirements. For example, those in service B were constrained by the need to deliver gym-based activities, whereas those in services A and C operated from various community bases and were able to accompany clients to other agencies or activities if required. Health trainers in services A and C were more likely to have personal reasons for taking up the role and often used their own experiences when working with clients:

*I know what it's like to be struggling. So you're not dictating to them and saying “Well, you shouldn't have had that cream cake”, you know. I've been there, I've done it and I've eaten the same cream cake. […] I think that's a big difference, because I've been through the system where I've been lectured at by people that just sit on the other end of a table and lecture you, and it doesn't work.* (Health trainer A4.0)

Managers in all three services reported that the health trainers were committed to their communities and enthusiastic about the role, and that they were recruited for those reasons. Although the teams were diverse in terms of their personal and professional backgrounds, it was felt (by staff and clients) that inter-personal skills were more important than formal qualifications:

*I think you can have 1,000 qualifications but if you're no good at your job or if you… You need to be able to speak to people and have time with people and listen to people. And if you can't and you've got all the qualifications in the world, you're still hopeless, really, for this job.* (Health trainer B8.0)

### Self-reported outcomes

As a consequence of heterogeneity in their aims and approaches, the three services also differed in terms of the effects that were perceived to result from the interventions. Clients described a range of self-reported behaviour changes and other outcomes, which are presented below.

#### Knowledge gains

Clients from each of the health trainer services reported gaining knowledge about healthy lifestyles, particularly in relation to food and nutrition. In many cases, this knowledge appeared to have been retained at the 6 and 12-month follow-up interviews:

*You get to the stage where you sort of know what you can… what [food] you can have and, like, how much of it. Sort of a portion and whatnot. […] It's just sort of ingrained in now. It's there. You know what you're doing so you're not thinking, “Oh well, I can only have two carbohydrates here or two there.” It's just automatic.* (Client A3.6)

There were examples of individuals becoming more aware of health messages and also more conscious of their ‘unhealthy’ behaviours, suggesting they were making informed choices:

*I've got to get back into it [following the recommended lifestyle changes], you know. Cut out my chocolates and things. I've just bought some chocolate biscuits this morning, which I shouldn't have. But I feel guilty about it. At least I have the decency to feel guilty. I know I'm doing wrong.* (Client B5.12)

#### Health-related behaviour changes

Much of the client feedback related to dietary changes, ranging from cooking without salt to growing their own fruits and vegetables. An important outcome involved adopting more structured lifestyles; for example, going from eating one to three meals a day or planning meals in advance. This often involved more home cooking, trying new ingredients and changing their purchasing habits:

*I don't eat bread anymore and I haven't actually missed it, so… And if I, I don't eat sweets very often. But I've found I don't buy food – the wrong kind of food – I just don't buy it now.* (Client A2.3)

Clients were unwilling to make some changes; as one put it, “*you can't have mince and no dumplings*” (Client A3.3). Rather than asking people to give up certain foods, health trainers suggested healthier ways to prepare them or substitute them for an alternative. Participants described a cascade effect, whereby these changes often influenced the behaviours of friends and relatives:

*For children a good idea is we grated carrot [into the recipe] as well and liquidised it with a hand blender just a tiny bit. And do you know I made it for [name] – my grandchild – and she loved it. And she used to hate it, you know. And it made her eat things what she wouldn't have eaten. […] The people I've given the recipes to – because we came out with all the recipes of everything and how to do it just like in layman's language – yeah. It was brilliant.* (Client A14.6)

There were also examples of clients making and maintaining behaviour changes in relation to physical activity, with some previously sedentary individuals describing exercise as “*addictive*”. A key factor was finding enjoyable activities that fitted around their other commitments:

*I've been keeping up my salsacise. So Monday I'm back at work now, Tuesday I keep free for like doctors' appointments and things like that, Wednesday at work, Thursday's sewing, and Friday's going to be salsacise in the morning, meditation in the afternoon and then if I keep it up there could be belly-dancing in the evening. So I'm keeping myself busy and keeping, trying to keep myself active. But I've tried to do things that I enjoy rather than, you know, like exercise classes and things like that. I still can't get away with them.* (Client C6.6)

#### Physical health improvement

Improvements in existing health conditions were frequently reported by clients who had increased their physical activity levels with the support of a health trainer. For example, one client (B13) experienced fewer asthma attacks and was advised by his GP to reduce his angina medication following completion of a gym-based intervention. Others reported reduced blood pressure, fewer symptoms of irritable bowel syndrome, and improved blood sugar levels:

*I'm a diabetic and it has actually, it brought my sugar levels down because I was getting exercise. […] When I had my blood results – and even the dietician, she was so pleased – it had come from 9.1 or something to 8, you know. So she said, “Oh, keep it up”, which as I say, I'm going to.* (Client B24.3)

Engaging in regular exercise was also reported to bring benefits in terms of better sleeping patterns, enhanced mood, and improvements in muscle tone, function and mobility. Participants felt their fitness levels had increased and some had reduced their pain medication usage:

*I'll tell you what I'm finding – I'm bending down easier… I suddenly found I was in my cupboard for something and, eeh, I thought, I can get up here great, where before it was [makes groaning noise] (Laughs). […] And I'm on no painkillers now. Just Paracetamol now, as needed, where I was on Diclofenac. So that's good, isn't it?* (Client A14.12)

Due to the combined effects of increased physical activity and dietary changes, many clients reported significant weight loss following completion of health trainer interventions. One (C25) described feeling as if she had “*unzipped a fat suit*”. A minority had gained weight and often attributed this to an illness or new medication, while others had maintained their weight in spite of such developments. There were also examples of family members losing weight and feeling better as a result of consuming healthier foods and taking part in activities with their partner or parent.

#### Psychosocial benefits

Health trainers often became a key source of social and emotional support for clients. Clients who were seen as vulnerable, such as those with mental health problems or disabilities, tended to require additional input to build their confidence:

*Working with [client C15], it's been a slow process. Slow progress. And I have seen some changes. There's a lot more confidence and I'm really pleased. Encouraging him to go out and meet more D/deaf people. On weekends he goes out with his friends – he might go to the pub or watch the football match. […] So he seems like he's getting more involved with things, which is positive and that's really good. And I'm trying to encourage that.* (Health trainer C12.12)

Becoming more socially active was identified as an important outcome by several participants, who reported developing the confidence to attend activities such as walking groups, sewing classes and luncheon clubs. Those with limiting long-term conditions experienced significant benefits from having opportunities to interact with others, build new social networks and take part in activities that fostered independence. The following quote is from a client with progressive multiple sclerosis:

*Mentally it has done me the power of good. You don't feel so useless. You can go out and do something, and mentally that's brilliant. […] The gym is the one place I don't have to take [the wheelchair]. And mentally that's such a high really – a victory, a one in the eye for the disease – if that makes any sense. (Laughs)* (Client B8.3)

Many clients attributed enhanced self-esteem and coping skills to participation in these activities. Health trainers also benefitted personally from their formal training and learning ‘on the job’. One example involved a staff member who reported feeling better able to cope with her partner's alcohol problems as a result of the knowledge and contacts gained through her role as a health trainer.

### Factors influencing maintenance of lifestyle changes

Employing a longitudinal approach enabled us to examine outcomes in the short- and medium-term (i.e. over a 12-month period) and also identify challenges to their sustainability. In cases where behaviour changes had not been maintained, the explanations given by clients primarily involved the cost and timing of activities, ill-health or low mood. Some continued to smoke or eat calorific foods because they found these behaviours mood-enhancing and used them as coping strategies:

*I am more aware of portion size than what I was before. I do try to limit that, but it's… It's dependant on my mood, I must admit. When I'm… I call it my dark side comes out. Because it is really, really dark. I find that that's the worst time. And then I'm more easily triggered than normal people, for getting low, I must admit.* (Client C2.6)

Participants described temporary setbacks and particular times when they found it more difficult to follow a healthy lifestyle. In order to ensure that any behaviour changes could be maintained, clients felt it was important to have ongoing access to low cost services that were tailored to their needs. Health trainers also expressed reservations regarding the use of relatively short-term interventions:

*Interviewer: Do you feel [12 weeks] is the right time for you to be able to get people…?*

*Health trainer B8.0: I don't. No, I don't to be honest. Maybe 24 weeks – you know, give somebody a whole, like, half of a year to really make a good effort of it and then you can go back to the doctors and say, “Well, we tried six months' worth.” Because it seems… You might just be getting into your stride on three months and then it's time to call it a day.*

Several clients were not confident in their ability to sustain lifestyle changes without longer-term support from health trainers or peers as part of social networks formed through participation in health trainer-led interventions. The quote below highlights the potential for reversals in the health benefits experienced:

*I feel more cut off now than I ever have done. Because the people I met at the gym are lovely, but they've got their own lives as well. And I don't see them anymore. I keep in contact with a couple of them, you know, on a phone call. But it's not the same as somebody next to you saying, “Come on, you can do it.” And, “How are you?” It's not the same.* (Client B8.12)

In contrast, others described taking a break from their attempts to maintain lifestyle changes during busy periods in their lives. It was implied that these changes were more likely to be sustainable than previous attempts to adopt healthy lifestyles due to a change in perspective:

*When I first started out [with the health trainer service], if I missed the gym for a month that would have been it, I would have never gone back. But now I know I will so I don't worry over it. I know, oh, that's alright – once I get my head sorted and back to normal and I'm not as busy, I'll get back into it.* (Client C26.12)

It was common for clients to report feeling positive about the lessons they had learned during the health trainer interventions, but that often “*life gets in the way*” (B5) when it comes to implementing the advice. Staff were optimistic that equipping people with relevant information would lead to some benefit in the longer term, with one describing this as “*planting the seed*” (A2).

## Discussion

### Principal findings

Participants in this longitudinal qualitative study perceived the health trainer role as being effective in terms of supporting people living in socio-economically disadvantaged areas to make and maintain positive lifestyle changes. Outcomes reported by clients sampled from local health trainer services included weight loss or maintenance, improvements in existing health conditions, reduced medication usage and increased social activity. Many also described feeling more confident and better able to cope with difficult circumstances. A time lag often exists between the dissemination of health advice and associated behaviour changes, particularly in less affluent populations. However, our study identified several examples of lifestyle modifications being sustained in the short- (6 months) and medium-term (12 months). There were some cases where participants felt longer-term support was needed to maintain their behaviour change efforts. There existed a number of intrinsic and extrinsic threats to the maintenance of lifestyle changes, including low mood, ill-health, social isolation and accessibility (in terms of cost and timing) of health-promoting activities. Tailored, low cost interventions and services were felt to help in overcoming these barriers.

Our findings demonstrate that the ‘health trainer’ label applies to heterogeneous services with common traits that include efforts to address the underlying determinants of health and the delivery of person-centred interventions largely based on behaviour change theory. Differences in outcomes were linked to differences in the primary aims and activities of health trainer services; an exercise-on-referral model for those with existing conditions (service B) was more likely to yield physical health improvements, whereas a community development approach targeting those in difficult social circumstances (service C) focused on improving confidence and reducing social isolation. However, all three services worked with clients from disadvantaged or marginalised communities, including those with limiting long-term conditions and disabilities. Participants described benefits gained from taking part in enjoyable activities that fostered independence, allowed them to develop new social networks and fitted into their existing lifestyles. In some cases, these benefits were passed on to family members and friends, potentially increasing the likelihood of longer-term maintenance. Conversely, services that were withdrawn without appropriate support structures in place left the most vulnerable clients feeling isolated and unable to maintain their lifestyle changes alone.

### Results in context

Previous research demonstrates the diversity of lay health advisor (LHA) programmes in terms of aims, content and outcomes [18]–[20]. Where reviews have identified positive effects from such programmes, these have been most prominent in terms of increasing access to care for underserved populations [34], [35]. There is modest evidence to suggest that LHA interventions can be effective in improving knowledge on disease prevention, cancer screening and diabetes, but evidence relating to behaviour change is mixed [20], [36]. In contrast to our findings, there is little evidence to support the use of LHAs in general health promotion, particularly in relation to diet or physical activity [18]. Areas where the best available evidence suggests that LHA interventions are effective and cost-effective include: smoking cessation, tuberculosis treatment, management of chronic conditions, education to reduce neonatal and maternal mortality, and HIV prevention [18], [37]–[41]. We are not aware of any published studies relating to quantifiable changes in social connectedness as a result of participation in lay-led health improvement programmes, although there is an established evidence base on the role of social networks in chronic illness self-management [42], [43].

Qualitative studies suggest that service users value the natural helper and bridging roles of LHAs and appreciate their flexible, user-centred approach [44]. LHAs are seen as credible, valuable sources of information that may not be available or accessible elsewhere [45]. Where users report negative perceptions or experiences, these tend to be due to concerns about confidentiality or feeling uncomfortable in talking about certain issues outside the family setting [46]. We identified additional concerns regarding the use of time-limited interventions to promote long-term lifestyle changes and the resulting feelings of isolation that may result once this support is withdrawn. Studies generally report a range of health and social outcomes perceived to result from LHA interventions. In a six-year follow-up study of an asthma self-management programme with a rural Maori community in New Zealand, participants reported altering their smoking, exercise and dietary habits, as well as making changes that affected the whole family, such as having smoke-free homes [47]. They also reported being better able to communicate with health services, being more involved in a range of social and recreational activities, and having strengthened links with other community members. Reported outcomes from similar LHA programmes include enhanced self-esteem, raised awareness of health issues and local services, and increased access to health-promoting resources [48], [49].

While it is important to acknowledge other models based on lay or peer support, further evidence is needed in recognition of the unique features of the health trainer role. For example, many LHA programmes rely on volunteers or sessional workers, whereas health trainers are paid employees generally operating in the context of the English NHS. A recurring theme in the literature involves the complex and challenging issues faced by health trainers, such as abuse, alcoholism and debt [50]. Clients often present multiple physical, mental and social needs that are revealed over time and only once a trusting relationship has been established [51], [52]. Studies highlight the appeal of the one-to-one approach of most health trainer interventions and having sufficient time to discuss any issues with an impartial but approachable stranger [51]. A key issue, reiterated in our study, is avoiding a ‘one-size-fits-all’ approach. Few robust evaluations have assessed the outcomes of health trainer interventions. However, those undertaken to date demonstrate that health trainers can be successful in reaching clients from less affluent groups and supporting them to achieve their behaviour change goals, particularly weight loss [53], [54]. Local evaluations have identified a ‘ripple effect’ in terms of benefits for friends and family members, as well increased confidence levels and a sense of wellbeing for clients [55], [56]. Furthermore, economic evaluation of a health trainer service in Liverpool identified an incremental cost per quality adjusted life-year (QALY) of £14,480 for the intervention [57]. This is more favourable than the threshold of £30,000 per QALY set by NICE for NHS interventions, suggesting that health trainers can represent value for money.

### Strengths and limitations

The 44 participants in our study may not fully represent the diversity of health trainer service staff and clients. In particular, men and ethnic minority groups were under-represented. However, our sample encompassed a range of demographic, social and health backgrounds, and the gender and ethnic make-up reflects the profile of health trainer services locally. By including three heterogeneous services we have provided a fuller picture of the spectrum of health trainer interventions than studying one model in isolation, while emphasising the commonalities within the wider health trainers initiative. A major strength of our study was the use of a longitudinal approach, which allowed us to examine factors influencing maintenance of the reported outcomes. The use of serial interviews is particularly suitable for research that aims to understand evolving and complex processes [58]. Our sample decreased over time (figure 4), although the level of attrition was not as great as anticipated and the maintenance of the user sample was a particular strength.

Various strategies were used to enhance the credibility and reliability of our interpretations, including techniques associated with grounded theory which helped to ensure that emergent concepts were grounded in the data [24]–[26]. The use of NVivo facilitated line-by-line analysis because it allowed for the creation of nodes that provided storage areas for references to coded text [59]. At the final interview, participants were asked to review a summary of our discussions; feedback demonstrated that these narratives accurately represented what was said and were true to their lived experiences. It is not known what impact, if any, was maintained beyond the 12-month follow-up period. However, this represents the first longitudinal qualitative study involving health trainers and their clients – and the most comprehensive study of the initiative to date – enabling participants to articulate their views and experiences of participating in lay-led lifestyle interventions.

### Implications and future research

This study demonstrates the perceived impact of health trainers in terms of behaviour change and other health-related outcomes. They are neither a cheap option nor a quick fix, but they have a potential role to play in addressing health inequalities. The recent King's Fund report on clustering of unhealthy behaviours refers to health trainers as “an under-used and ready-made workforce to help drive the reduction of multiple lifestyle risks”, and suggests that, “In theory, this is exactly the sort of service that could have a big impact” (p.20) [60]. Others have suggested that a more systematic and comprehensive approach is needed, calling for greater integration of health trainers with primary care to avoid missed opportunities, inefficiencies and duplication [43]. Commissioners should consider funding health trainer services to work with local communities and deliver holistic, person-centred lifestyle interventions. The degree of heterogeneity within the initiative has been demonstrated. Commissioners should therefore use the available evidence to select the intervention models that best meet the needs of their local populations. Service providers can support health trainers and similar LHAs by providing adequate supervision and training that equips them with expertise in relevant behaviour change techniques, including relapse prevention. It is essential that providers think carefully about appropriate exit strategies, particularly if clients are socially isolated or in poor health. Dealing with complex, emerging issues highlights a need for training and development opportunities to be provided on an ongoing basis.

Concerns have been expressed about the strength of the evidence to support health trainers [41]. Limitations include a lack of rigorous research evaluating the impact of the health trainer role, the extent to which it leads to health improvements for clients and whether it is cost-effective. Our research has helped to identify relevant outcomes, identified constraints in the delivery of services, and examined the maintenance of lifestyle changes. Further research is needed to determine the effectiveness, cost-effectiveness and equity of health trainers and similar lay-led interventions. A rigorous, definitive outcome and economic evaluation with embedded process evaluation should be undertaken with an appropriate design that allows for the complexity and wide range of health trainer models delivered nationally. Alongside such a study, it is essential that robust monitoring and evaluation frameworks are put into place from the earliest stages of any new lay-led health improvement programme in order to assess and measure the most appropriate outputs, outcomes and impacts. Such a programme of research will help to inform the evidence-based commissioning of health trainers and similar services.
